# An improved multi-scale gradient generative adversarial network for enhancing classification of colorectal cancer histological images

**DOI:** 10.3389/fonc.2023.1240645

**Published:** 2023-11-13

**Authors:** Liwen Jiang, Shuting Huang, Chaofan Luo, Jiangyu Zhang, Wenjing Chen, Zhenyu Liu

**Affiliations:** ^1^ Department of Pathology, Affiliated Cancer Hospital and Institution of Guangzhou Medical University, Guangzhou, China; ^2^ School of Information Engineering, Guangdong University of Technology, Guangzhou, China; ^3^ Department of Pathology, Guangdong Women and Children Hospital, Guangzhou, China

**Keywords:** colorectal cancer, histological images, convolutional neural networks, generative adversarial networks, histological image synthesis, tissue type classification

## Abstract

**Introduction:**

Deep learning-based solutions for histological image classification have gained attention in recent years due to their potential for objective evaluation of histological images. However, these methods often require a large number of expert annotations, which are both time-consuming and labor-intensive to obtain. Several scholars have proposed generative models to augment labeled data, but these often result in label uncertainty due to incomplete learning of the data distribution.

**Methods:**

To alleviate these issues, a method called InceptionV3-SMSG-GAN has been proposed to enhance classification performance by generating high-quality images. Specifically, images synthesized by Multi-Scale Gradients Generative Adversarial Network (MSG-GAN) are selectively added to the training set through a selection mechanism utilizing a trained model to choose generated images with higher class probabilities. The selection mechanism filters the synthetic images that contain ambiguous category information, thus alleviating label uncertainty.

**Results:**

Experimental results show that compared with the baseline method which uses InceptionV3, the proposed method can significantly improve the performance of pathological image classification from 86.87% to 89.54% for overall accuracy. Additionally, the quality of generated images is evaluated quantitatively using various commonly used evaluation metrics.

**Discussion:**

The proposed InceptionV3-SMSG-GAN method exhibited good classification ability, where histological image could be divided into nine categories. Future work could focus on further refining the image generation and selection processes to optimize classification performance.

## 1 Introduction

Colorectal Cancer (CRC) is regarded as one of the most important malignant gastrointestinal cancers. Owing to the high incidence and mortality rates, CRC is also the second most common cancer in women and the third most common cancer in men. Despite differences in geographical distribution, age and gender, the global incidence of CRC is expected to increase by 80% by 2035. Most CRCs are sporadic (7080%), while about one-third of CRCs have a genetic component (1–3). Fortunately, early detection, correct diagnosis and appropriate treatment can effectively improve the survival of patients with CRC. Many studies have shown that the development of CRC can be effectively determined by histological image analysis such as a more accurate classification of histological images (4, 5).

Over the years, researchers have developed a wide range of methods based on Artificial Intelligence to accurately classify medical images (6–9). Traditional machine learning approaches for classifying images contain several steps, which are data preprocessing, manual feature extraction, manual feature selection, classification and so on. However, these approaches require prior domain knowledge and may not generalize well on test data (10).

Deep neural networks have been proposed and can be used for the classification of images. Specifically, convolutional neural networks (CNNs) are multilayered and trained with a back-propagation algorithm to classify. In medicine, CNNs are used to classify images to predict clinical parameters and outcomes, and have attained huge success (11, 12). Zhou et al. (13) introduced a new attention mechanism into CNN to classify the differentiation types of histopathological images of colorectal cancer. Kumar et al. (14) proposed a lightweight and less complex CNN framework to improve the classification performance of colorectal tissue. Khazaee et al. (15) developed a hybrid structure based on deep TL networks and ensemble learning to detect colorectal cancer.

However, supervised CNN training often requires a large number of expert annotation data to achieve high accuracy. In addition to this, only a small set of labeled data is available in many practical applications due to annotation costs and privacy issues. Additionally, labels are often unbalanced between grading and subtypes (16). Thus, an optimal solution is to develop a generative model to eliminate the issues mentioned above, which means that the sample size is increased through instances of the original data.

Traditional transformations like flipping, mirroring, scaling, and cropping are the most common image augmentation strategies. However, they do not really introduce new images with additional information and do not better fill the entire data distribution (16). Data augmentation with these simple transformations may not effectively enhance classification performance. In order to achieve more robust performance, a large amount of annotated and high-quality training data is usually required.

Recently, generative adversarial networks (17) (GANs) are increasingly active and widely used in medical data synthesis because of their excellent data generation capabilities without explicitly modeling probability density functions (16, 18–21). Augmenting existing medical images can significantly increase the sample size of the training set. It partly alleviates the problem of limited sample sizes of medical images due to inherent limitations such as imaging costs, tag costs and patient privacy (22, 23).

A typical GAN comprises a generator (G) and a discriminator (D) which are embedded in a competitive process. The discriminator is expected to perform accurate binary classification of real/fake images. The generator expects to fake images which are sufficiently realistic that the discriminator cannot accurately classify them. The two components are trained iteratively, and the performance increases alternately. As a result of the game competition, the generator’s performance can be significantly promoted.

However, it is of note that traditional GANs suffer from two prominent problems which are mode collapse and training instability. Mode collapse occurs when the generator captures only a subset of the data distribution, resulting in a lack of diversity in the generated samples. And the reason for the training instability of GANs is that when there is no overlap between the real and fake data distributions, the gradients passed from the discriminator to the generator become uninformative (24, 25). A number of GAN-variants have been proposed to alleviate these two problems, such as Deep Convolution Generative Adversarial Networks (DCGAN) (26), Self-Attention Generative Adversarial Networks (SAGAN) (27) and Multi-Scale Gradients Generative Adversarial Networks (MSG-GAN) (25).

Multi-Scale Gradients GAN (MSG-GAN) has been developed for the production of higher resolution images (25). This architecture utilizes the idea of progressive neural networks first proposed in (28) which starts with low resolution 4 *×* 4 pixels image and begins to grow with the training progressing for the generator and discriminator. MSG-GAN alleviates the instability problem by allowing the flow of gradients from the discriminator to the generator at multiple scales to generate high-resolution images. Compared with PROGAN (29), latent spaces of the generator and the discriminator in MSG-GAN are connected so that more information is shared between the generator and the discriminator. To be specific, multi-scale images are sent to the discriminator and linked to the corresponding main path. The discriminator can not only view the final output of the generator but also the outputs of the middle layers. Therefore, the discriminator becomes a function of the generator’s multiple scale outputs, and importantly, it passes gradients to all the scales simultaneously.

MSG-GAN can synthesize more realistic and diverse samples, so we adopt it as the basic framework. However, the images generated by GANs may have label ambiguity, to be specific, a generated image of one class is misclassified into another class, which degrades classification model performance (16). Our goal in this paper is to enhance the classification performance, thus we apply a selection mechanism to filter the synthetic images, guaranteeing that all selected generated images are more credible and can be classified into some class with certainty.

In this work, we propose a Selective GAN model based on MSG-GAN (SMSG-GAN) to generate high-fidelity images. Furthermore, we also employ additional GANs such as DCGAN and SAGAN for comparison. In order to obtain more realistic and convincing images, a selection mechanism is proposed to screen images, which takes advantage of a trained model to filter the generated images, and the images with higher predicted probability than the threshold are saved. We both qualitatively and quantitatively evaluate the proposed SMSG-GAN by performing colorectal tissue image classification with an advanced CNN classifier trained on augmented data and applying various evaluation metrics. Experimental results show that SMSG-GAN can effectively enhance classification performance and its superior generation ability.

## 2 Materials and methods

### 2.1 Colorectal cancer data

**Figure 1:**
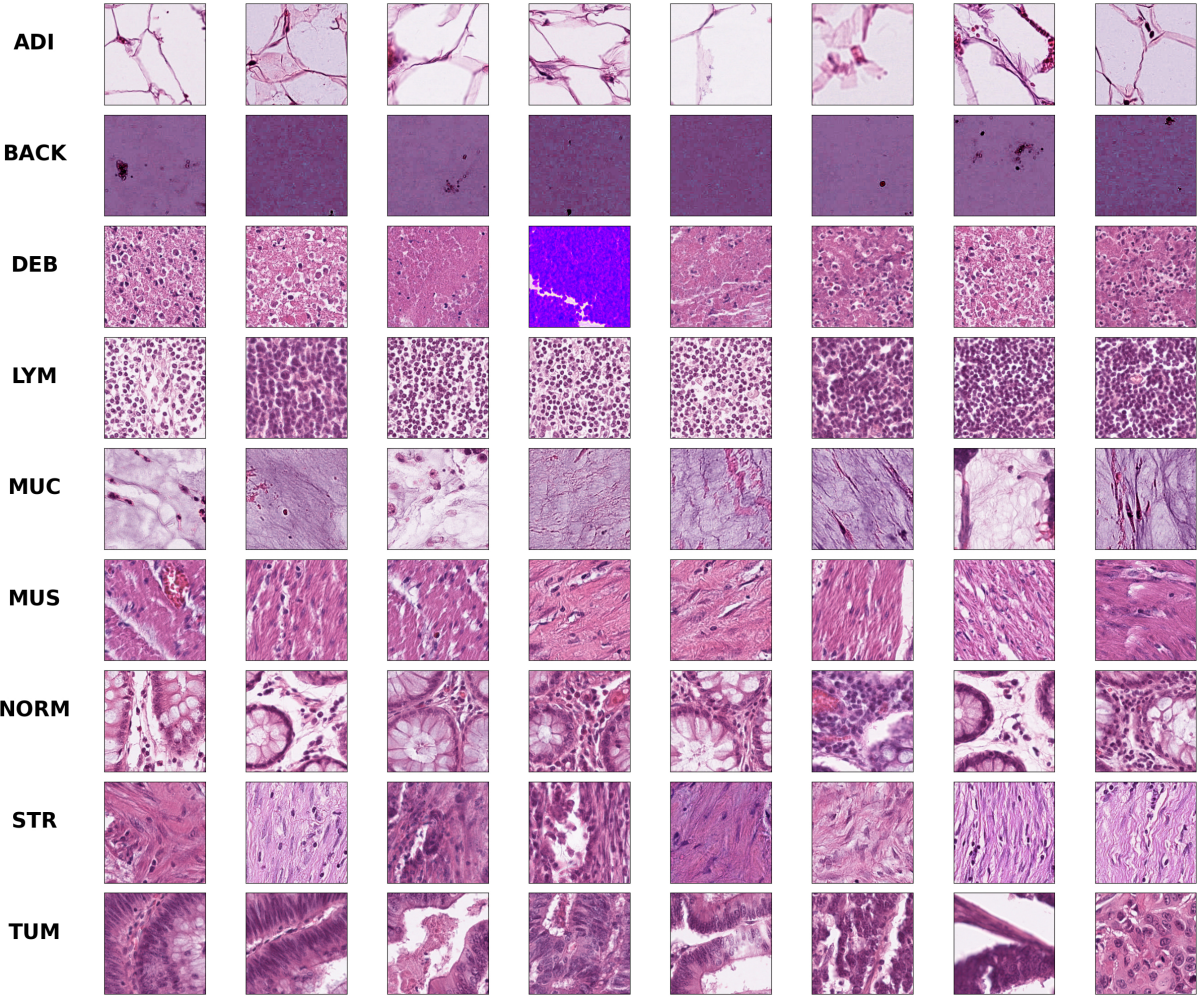
Example images for each of the nine tissue classes represented in the NCT-CRC-HE-100K data set.

Colorectal cancer is a type of solid tumor as well as a complex disease. Additionally, Colorectal histological images generally contain a variety of tissue types and features, which makes it extra complicated to analyze. Hematoxylin-Eosin (HE) stained histological images are the main tool used to diagnose CRC and determine the stage of CRC. In HE slides of CRC patients, it is vital to differentiate normal tissues from tumor regions (30). In this paper, we use the open-access histological data set of nine tissue classes from NCT-CRC-HE-100K for data augmentation and tissue classification. The data set is provided by Kather et al. (3), which is manually delineated single-tissue region in 86 CRC tissue slides, generating more than 100,000 HE image patches. There are nine categories including adipose tissue (ADI), background (BACK), debris (DEB), lymphocytes (LYM), mucus (MUC), smooth muscle (MUS), normal colon mucosa (NORM), cancer-associated stroma (STR) and colorectal adenocarcinoma epithelium (TUM). Images randomly selected from each category of this data set are illustrated in **Figure 1**. We evaluate the accuracy of tissue classification with an external validation set: the NCT-VAL-HE-7K data set, which contains 7180 image patches from 25 whole-slide images. The dimensions of all the images are 224 *×* 224 *×* 3 and cropped at a magnification of 20*×* (0.5*µm/pixel*). We used all samples in the NCT-CRC-HE-100K and NCT-VAL-HE-7K datasets.

**Figure 2:**
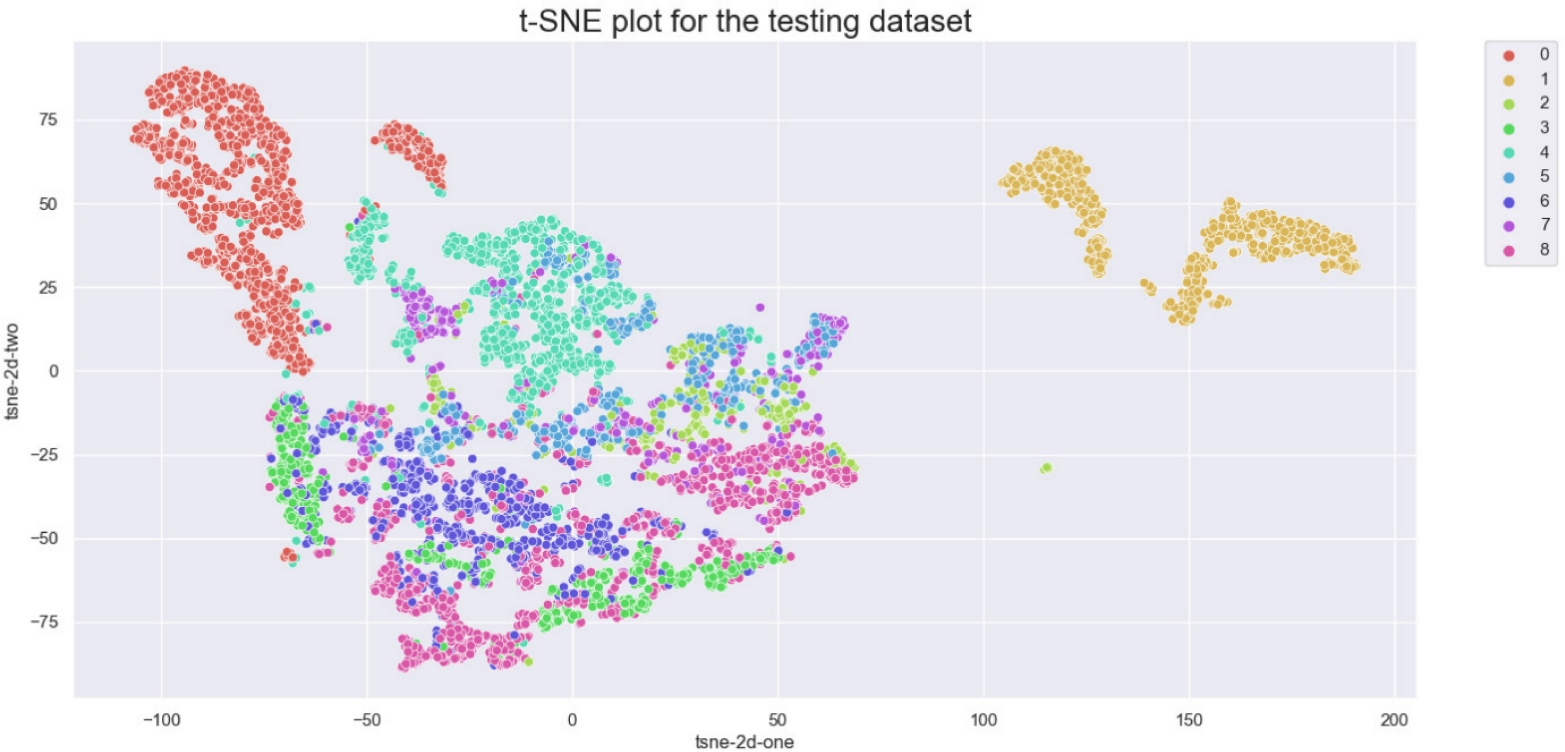
t-SNE of the testing set. Classes: 0=ADI, 1=BACK, 2=DEB, 3=LYM, 4=MUC, 5=MUS, 6=NORM, 7=STR, 8=TUM.

The aim of this study is to achieve maximum separation between different classes in the classification process. To this end, we utilize t-distributed stochastic neighbor embedding (t-SNE) to visualize the class separation of the NCT-VAL-HE-7K dataset (31). t-SNE is a nonlinear dimensionality reduction technique that maps high-dimensional data into twodimensional or three-dimensional space for visualization purposes. The results of the visualization are presented in **Figure 2**. Most tissue classes are found to cluster together except for ADI and BACK classes. This could be due to the fact that ADI and BACK classes contain less tissue, making them easier to distinguish from other classes.

### 2.2 Proposed methodology

**Figure 3:**
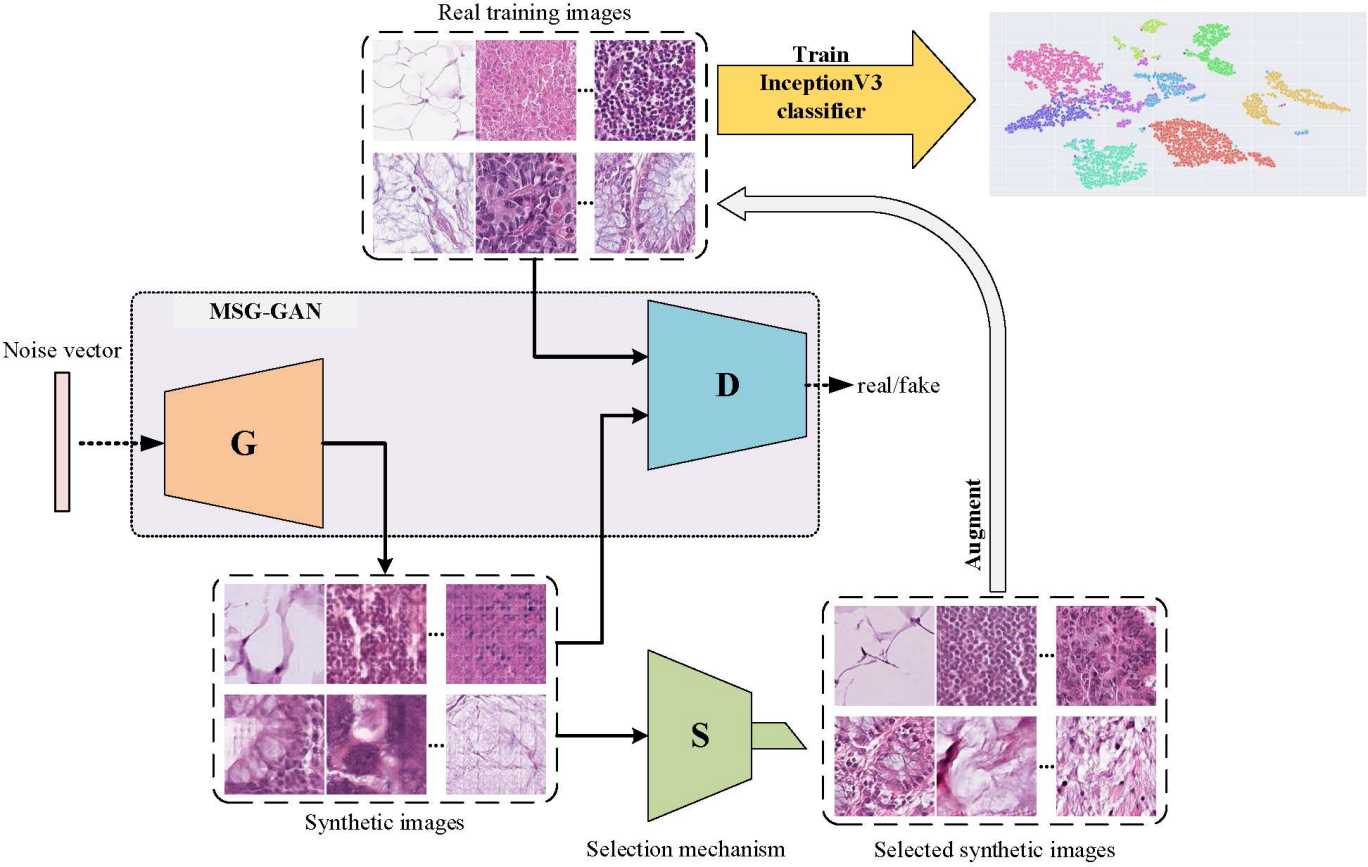
Flowchart of InceptionV3-SMSG-GAN.

**Figure 4:**
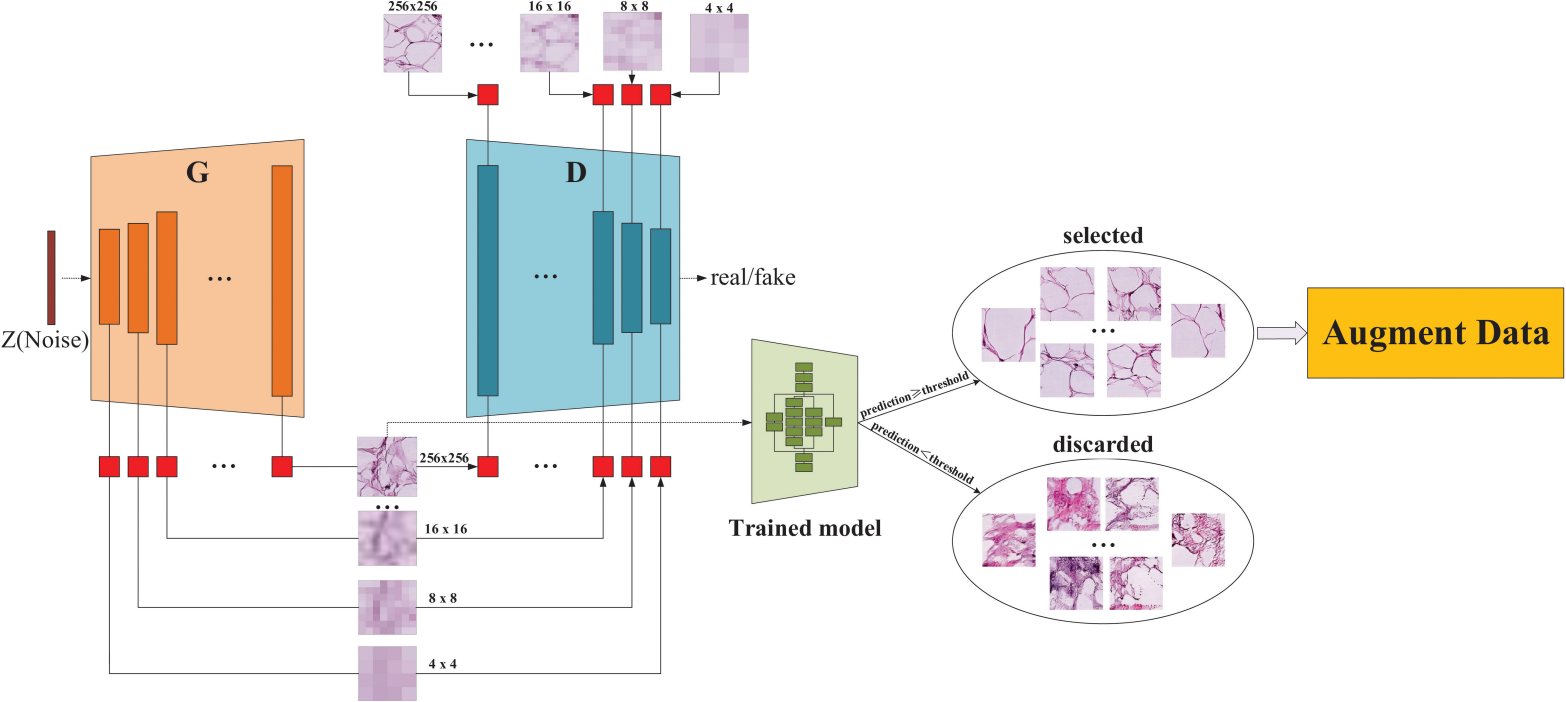
The architecture of the proposed SMSG-GAN. Each orange bar in the generator represents each convolutional block of the generator, similarly, each indigo blue in the discriminator represents each convolutional block of the discriminator, and the red block represents a convolutional layer with a kernel size of 1 *×* 1.

An overall flowchart of the proposed method named InceptionV3-SMSG-GAN is depicted in **Figure 3**. InceptionV3-SMSG-GAN is presented to improve the classification performance of colorectal cancer histopathological images. To be specific, we take advantage of MSG-GAN to generate a large amount of training samples for the downstream classification task, and then use a selection mechanism to filter the synthesized data. After the selection, the selected data and the original training set are put together into the classification network as the training set and validation set. Taken together, the proposed approach contains two parts which are selective data augmentation and downstream classification task. Among them, the selective data augmentation methodology consists of two stages, namely data generation and selection, as shown in **Figure 4**. Note that the SMSG-GAN method is adopted for each class in turn, here displays the generation for the ADI class. In the following subsections, detailed information about each of these stages is provided.

#### 2.2.1 Data synthesis

As illustrated in **Figure 4**, the proposed SMSG-GAN framework trains with progressive growth resolution. The intermediate layers of the generator share features with the corresponding intermediate layers of the discriminator, which can improve the training efficiency and quality of the generated images. This sharing promotes information flow between the generator and the discriminator, aiding the generator in better understanding the data distribution and features, and producing more realistic images. Besides, sharing features can reduce the number of parameters in the models, preventing issues such as overfitting from occurring. The generator of SMSG-GAN outputs generated histopathological images, while the discriminator outputs the probability that the given image is real. Furthermore, the generator and the discriminator in SMSG-GAN consist of seven convolutional blocks each, enabling them to generate images of varying sizes simultaneously, resulting in higher-quality images. It is of note that only images with the highest resolution of 256 *×* 256 pixels are adopted. Moreover, the optimization of SMSG-GAN is performed with respect to a joint loss function for *D* and *G* as given in Equation (1).


(1) $$\left\{ \begin{array}{l} l_{dis}\left(x_{real},\;x_{fake}\right)=E\left[R\left(1-rf\right)\right]\;+E\left[R\left(1+fr\right)\right]\\ l_{gen}\left(x_{real},\;x_{fake}\right)=E\left[R\left(1+rf\right)\right]\;+E\left[R\left(1-fr\right)\right] \end{array}\right.$$

where, $rf=D\left(x_{real}\right)-E\left[D\left(x_{fake}\right)\right]\mathrm{,f}r=D(x_{fake})-E\;[D(x_{real})]\mathrm{,R}\left(x\right)\;=\;max\left(x,0\right)$

where *l* ^dis^ (*x*_real_ *,x*_fake_) and *l*_gen_ (*x*_real_ *,x*_fake_) are the loss of discriminator and generator, respectively. *x*_real_ represents real images from training data, and *x*_fake_ denotes fake images that generator synthesizes.

We utilize a noise vector of 256 dimensions from a standard normal distribution in the initialization phase. The latent vector is used as the input of a generator to generate images of different sizes through a series of layers, and the generated images and real images of their corresponding scales are fed into the discriminator which ultimately estimates the reality of the images. A two time-scale update rule ensures that training reaches a stationary local Nash equilibrium if the discriminator learns faster than the generator (32). In light of this, we apply different learning rates for the generator and the discriminator to achieve better performance. The generator trained for the nine classes of tissue images is trained with Adam (lr=0.0001), and the learning rate of the discriminator is 0.0004. Other settings in synthesis experiments are as follows, all models are trained with the net budget of 100 epochs and the batch size is set as 8. By applying the above configurations, SMSG-GAN trains for each class of colorectal tissue separately. After the training is completed, 5,000 images are generated for each class, increasing the amount of data for the downstream classification task.

During the synthesis experiment, DCGAN and SAGAN are also utilized to generate images using the NCT-CRC-HE-100K data set for further generation comparison. Compared with the original GAN, DCGAN makes some changes to CNN architecture to improve the quality of samples and convergence speed. The main improvement is to replace the pooling layers in the discriminator with deconvolution and fractional deconvolution in the generator (26). The size of generated images in (26) is 64 *×* 64 pixels, so we modify the number of the channels in the architecture to synthesize the same size as the output images of SMSG-GAN. As for SAGAN, self-attention module and spectral normalization (33) have been used in both generator and discriminator to enhance performance. The self-attention module is used to compute the response at one location as a weighted sum of features at all locations for a better balance between the ability to model long-range dependencies and statistical efficiency. We also change the number of channels in convolutional layers in the SAGAN architecture and make some modifications to generate images with a resolution of 256*×*256 pixels. It is of note that the same configurations as SMSG-GAN are adopted in the generation experiments of DCGAN and SAGAN.

#### 2.2.2 Selection mechanism for synthetic data

In order to make better use of training data and alleviate the problem of overfitting during training, data augmentation has become a commonly utilized method for deep neural network training. The goal of data augmentation is to augment the original training set with new samples that follow the original data distribution. Therefore, a good data augmentation scheme should generate samples that follow the original data distribution but are different from the original training set. Conversely, a poor data augmentation scheme will produce samples that deviate from the original data distribution, thereby misleading training.

Most data augmentation methods based on GAN directly add generated images to the training set, which results in the varying quality of synthetic images affecting the effectiveness of augmentation. Accordingly, for the sake of a high-quality synthetic data set, a filtering mechanism is proposed to make sure that the augmented samples satisfy the original data distribution. Specifically, we use a GoogleNet InceptionV3 CNN model (34) trained without any data augmentation on nine categories of colorectal tissue images, which also serves as a baseline model in classification experiments, to predict the class probabilities of synthetic images. After the training, we select generated images with class probabilities predicted by the trained model greater than a given threshold *α*.

Regarding the choice of *α*, we provide an ablation study in Section 3.3. The selected images can be confidently classified into certain classes and thus contain sufficient diagnostic features. In order to benefit improvement of classification performance, the selectively generated images are added to the original data for training.

#### 2.2.3 Colorectal tissue images classification

For classification experiments, we utilize the InceptionV3 model for classification with the nine classes mentioned in Section 2.1. During training, we replace the last fully connected layer presented in the original network architecture with two fully connected layers which are followed by a rectified linear unit activation (ReLU) activation function and a dropout layer with 0.5 probability. The ReLU function is a common type of nonlinear activation that maps negative values to zero and returns the original value for positive values. The final output of the model is the probability of the category of the histopathological image. The main parameters are set as follows: batch size is 64 and epochs is set as 20. It is also worth noting that the model is trained using stochastic gradient descent with an initial learning rate of 0.001 after defining the network structure. During the training process, we implement a changeable learning rate scheme to further enhance classification performance:


(2) $$lr_{ep}=lr_{ep-1}\times [\frac{1+cos\left(\pi \times ep/epochs\right)}{2}\times \left(1-lr\;f\right)+lr\;f]$$

where *lr* represents the learning rate, and *ep* is the number of epochs, we set *epochs* and *lr f* to 20 and 0.1, respectively.

#### 2.2.4 Statistical analysis

In order to further demonstrate that the classification results of the proposed method are not obtained by chance, we utilize the Paired t-Test. We evaluate the significant difference between the proposed method and the baseline by calculating the P values of the Paired t-Test for the classification overall accuracy which are obtained by the models. All the classification experiments are run three times with different random seeds, where the best classification performances for the two models are reported in **Table 1**.

**Table 1:** Classification performance of InceptionV3 model for each tissue class.

	Baseline(w/o data augmentation)				Proposed(with SMSG-GAN)***			
	Precision	Recall	Specificity	Overall Accuracy	Precision	Recall	Specificity	Overall Accuracy
ADI	0.926	0.85	0.984	0.8687	**0.984**	**0.894**	**0.997**	**0.8954**
BACK	0.955	1.0	0.994		**0.967**	**1.0**	**0.995**	
DEB	**0.722**	0.805	**0.985**		0.708	**0.879**	0.982	
LYM	0.961	0.965	0.996		**0.986**	**0.978**	**0.999**	
MUC	0.945	0.871	0.992		**0.963**	**0.921**	**0.994**	
MUS	0.606	0.833	0.951		**0.673**	**0.865**	**0.962**	
NORM	0.818	0.869	0.978		**0.838**	**0.880**	**0.98**	
STR	0.796	0.390	0.994		**0.815**	**0.430**	**0.994**	
TUM	0.903	0.945	0.979		**0.908**	**0.949**	**0.980**	

The left side denotes the classification results of InceptionV3 model without data augmentation, which serves as a baseline. while the right side represents the results of the proposed method which utilizes SMSG-GAN to augment data and then uses InceptionV3 model to classify tissue types. *** denotes significant difference.

The bold values indicate the best performance.

Statistical analysis is done in Python (version 3.6) using stats module from Scipy library (version 1.5.4). P values< 0.05 are considered to be statistically significant. The P value of the Paired t-Test is shown in **Table 1**. Therefore, the proposed method has statistically significantly different classification performance with the P value of 0.001.

## 3 Results

In this section, we present the evaluation results of the proposed methodology. The first experiment was a comparison of classification training on colorectal tissue images. After the training of classification, we utilized an additional data set for the test. The second experiment used various GANs to generate the nine classes of colorectal tissue images. Furthermore, we also provided a performance comparison of DCGAN, SAGAN and SMSG-GAN on generating the nine classes of colorectal tissue images. The performance comparison was based on measuring the quality of the synthetic data through several commonly used metrics.

### 3.1 Results of classification

We used the InceptionV3 model without any data augmentation assistance as the basic classification baseline. As for our approach, selective synthetic data was added to the original training set for further classification experiments, we also utilized the same InceptionV3 model used in the baseline to classify the nine classes of colorectal tissue patches. During the classification training, we fixed the same parameters as the classification baseline model. When the training was completed, we utilized the NCT-VAL-HE-7K data set to evaluate the performance of the two models.

In order to evaluate the classification performance of the proposed method, we adopted confusion matrix, precision, recall, specificity, and overall accuracy. The confusion matrix represents the predicted labels of the classifier versus the true labels, larger values on the diagonal indicate better classification performance. As for other measures, a larger value means better prediction performance of the classifier.

**Figure 5:**
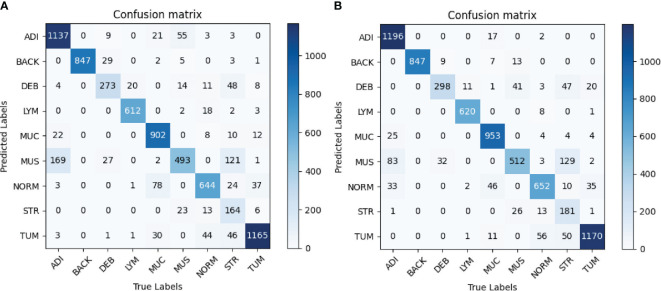
**(A)** Baseline. **(B)** Proposed method. Confusion matrices of the classification results using InceptionV3. **(A)** represents confusion matrix of the InceptionV3-based classification without any augmentation, while **(B)** represents confusion matrix of the InceptionV3-based classification with selective synthetic data using our proposed method.

The confusion matrices of the evaluation are shown in **Figure 5**. **Figure 5A** shows that the most obvious confusion existed between MUS and ADI. Furthermore, most misclassifications arose between the classes MUS and STR as well as between NORM and MUC. As illustrated in **Figure 5B**, the number of the class ADI misclassified to the class MUS decreased significantly, and more images belonging to the STR category and LYM category were correctly classified. Taken together, we attained higher values on the diagonal of the confusion matrix than the baseline.

**Table 1** depicts the precision, recall and specificity of each category, and overall accuracy for all categories in detail. The best results are in bold. In terms of precision, InceptionV3-SMSG-GAN yielded more competitive results than the baseline model in most categories except for DEB. In particular, the precision of the ADI and MUS classes increased dramatically by 5.8% and 6.7%, respectively. For recall metric, InceptionV3-SMSG-GAN displayed significantly better performance in all classes. The recall of the ADI, DEB, MUC, MUS and STR classes increased by about 4.4%, 7.4%, 5%, 3.2% and 4%, respectively. Additionally, the proposed method achieved better results than the baseline model for specificity evaluation metric except for DEB which decreased slightly by 0.3%. In summary, our approach achieved much higher overall accuracy with 89.54% and generally got higher values in most metrics than the baseline model. In most cases, the classification with SMSG-GAN led to better performance results than without augmentation of the training data, which demonstrated that SMSG-GAN was able to enhance the classification performance of the colorectal cancer tissue.

**Figure 6:**
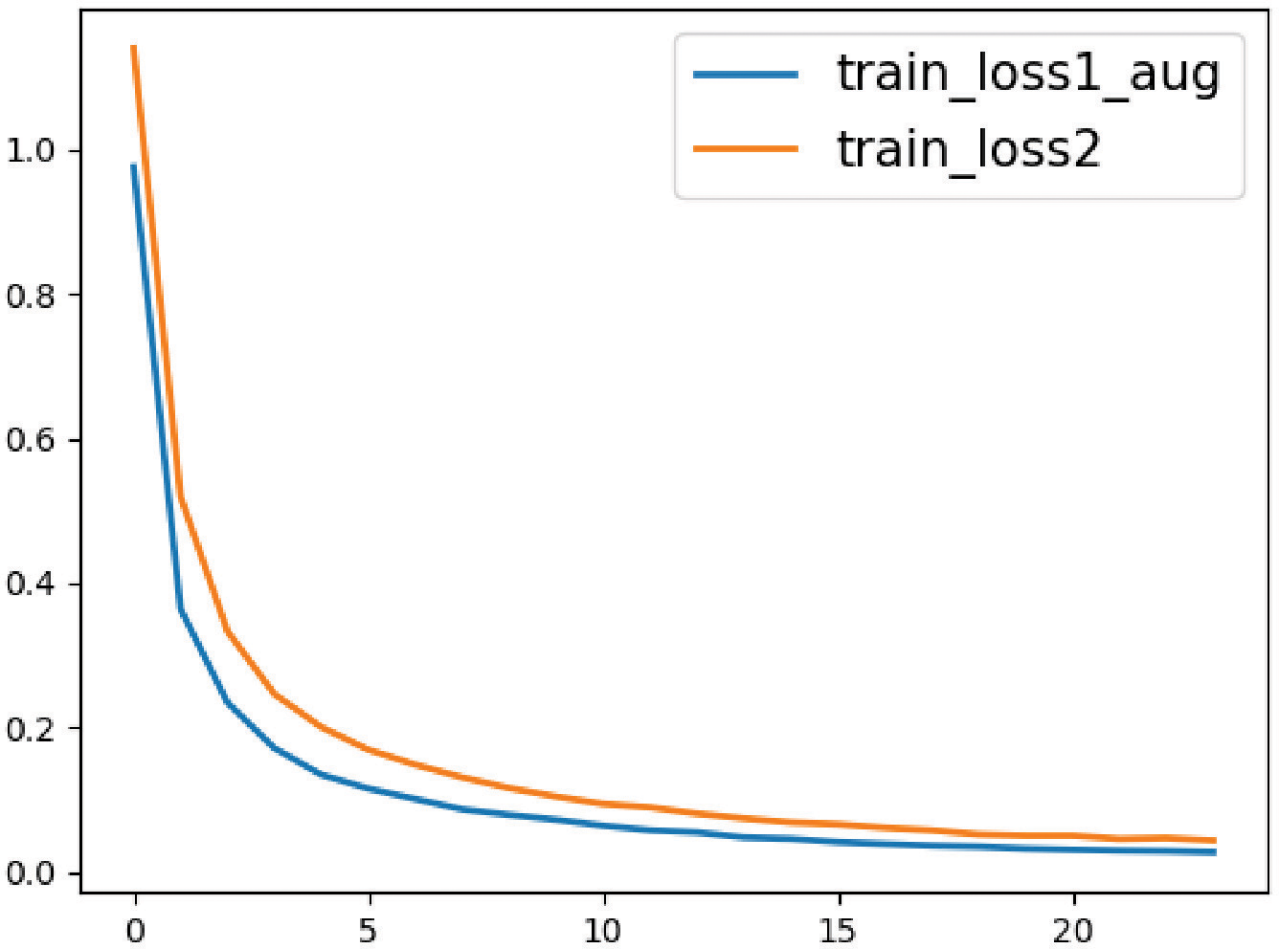
The loss value curves for the baseline and proposed method. The orange line represents the classification without data augmentation, and the blue line represents the classification with selective synthetic data.

During training both classification models of colorectal histological images, we monitored the training loss value which is depicted in **Figure 6**. The Training loss curve depicts convergence across epochs and demonstrates better anytime performance with the proposed approach in comparison to the baseline. The loss values of InceptionV3-SMSG-GAN are lower all the time, which demonstrated the significance of the proposed method and better convergence.

To further verify the effectiveness of the proposed method, ShuffleNetV2 and MobileNetV2 were applied for comparison. **Table 2** shows the overall accuracy of classification performance. The proposed method achieved the best overall accuracy for all categories, which is 0.0267 larger than the baseline model, 0.1038 larger than ShuffleNetV2, and 0.0476 larger than MobileNetV2.

**Table 2:** Classification performance of the proposed method and other algorithms.

Methods	Overall Accuracy
Baseline	0.8687
ShuffleNetV2	0.7916
MobileNetV2	0.8478
Proposed	**0.8954**

The bold values indicate the best performance.

**Figure 7:**
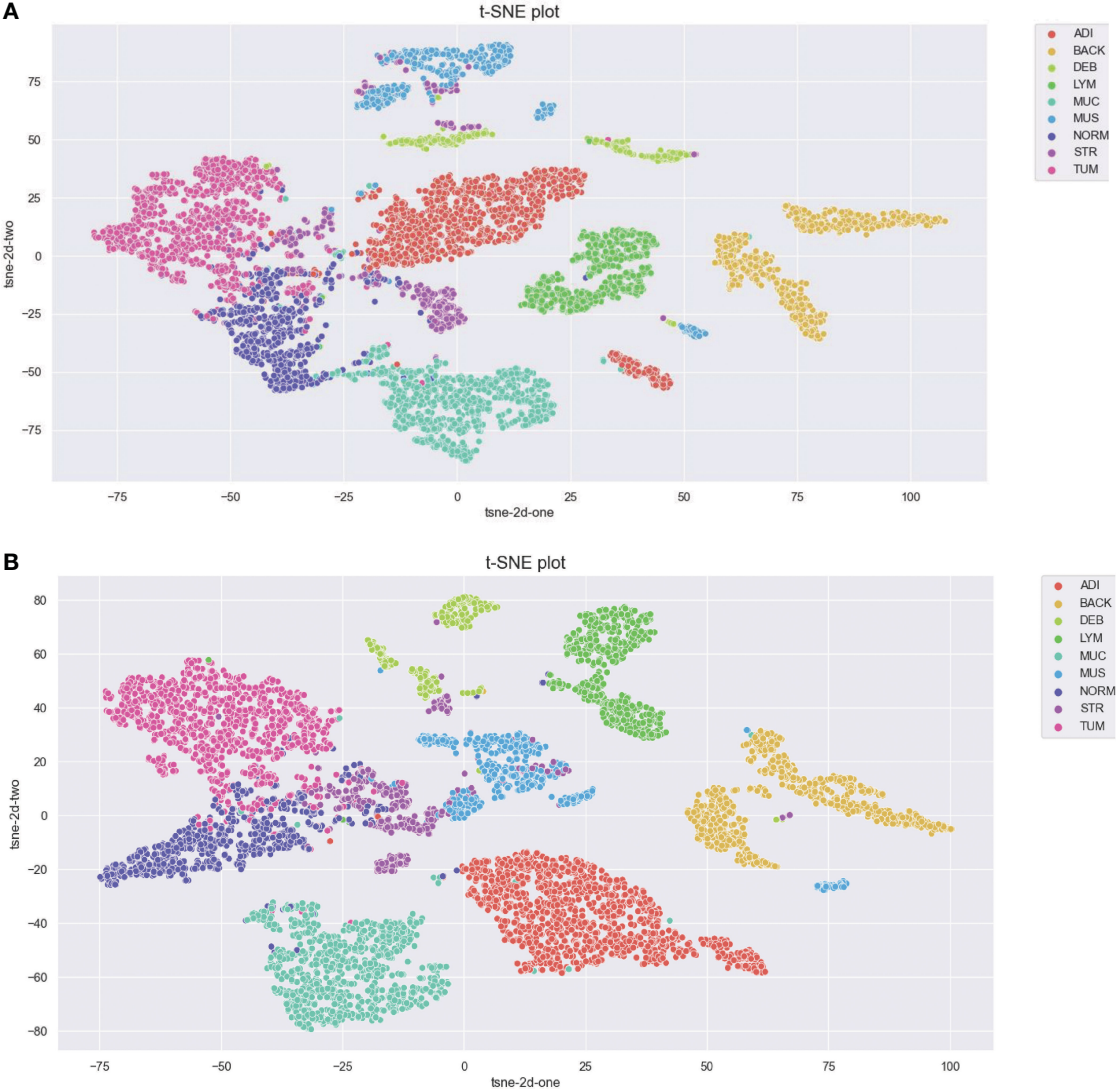
**(A)** Baseline. **(B)** Proposed method. Visualization of the class separation based on t-SNE of deep layer activations for the testing data. **(A, B)** represent t-SNE of the testing set based on deep layer activations of InceptionV3 (w/o data augmentation) and InceptionV3 (with selective synthetic data augmentation), respectively.

We further visualized the internal representations of tissue classes by using t-SNE on deep layer activation of both methods and saw a nearperfect separation of the classes in the testing set. **Figure 7** shows that the distributions of features extracted from the baseline InceptionV3 model for DEB and STR were much more concentrated, yet each category with InceptionV3-SMSG-GAN was more separate. This showed that the classification model with data synthesized by SMSG-GAN learns image features that allowed the separation of nine tissue classes, which outperformed the baseline model.

### 3.2 Results of synthetic colorectal tissue images

To facilitate a more sufficient training data set to train the classifier, we synthesized the colorectal tissue images using GANs. In the training of classification, SMSG-GAN generated 5000 images with a resolution of 256*×*256 pixels for each class of colorectal tissue images. Xue et al. (16) did some experiments which showed that the best augmentation performance was achieved when the augmentation ratio was 0.5, where the augmentation ratio represents the proportion of the number of generated samples to the number of original training samples.

**Figure 8:**
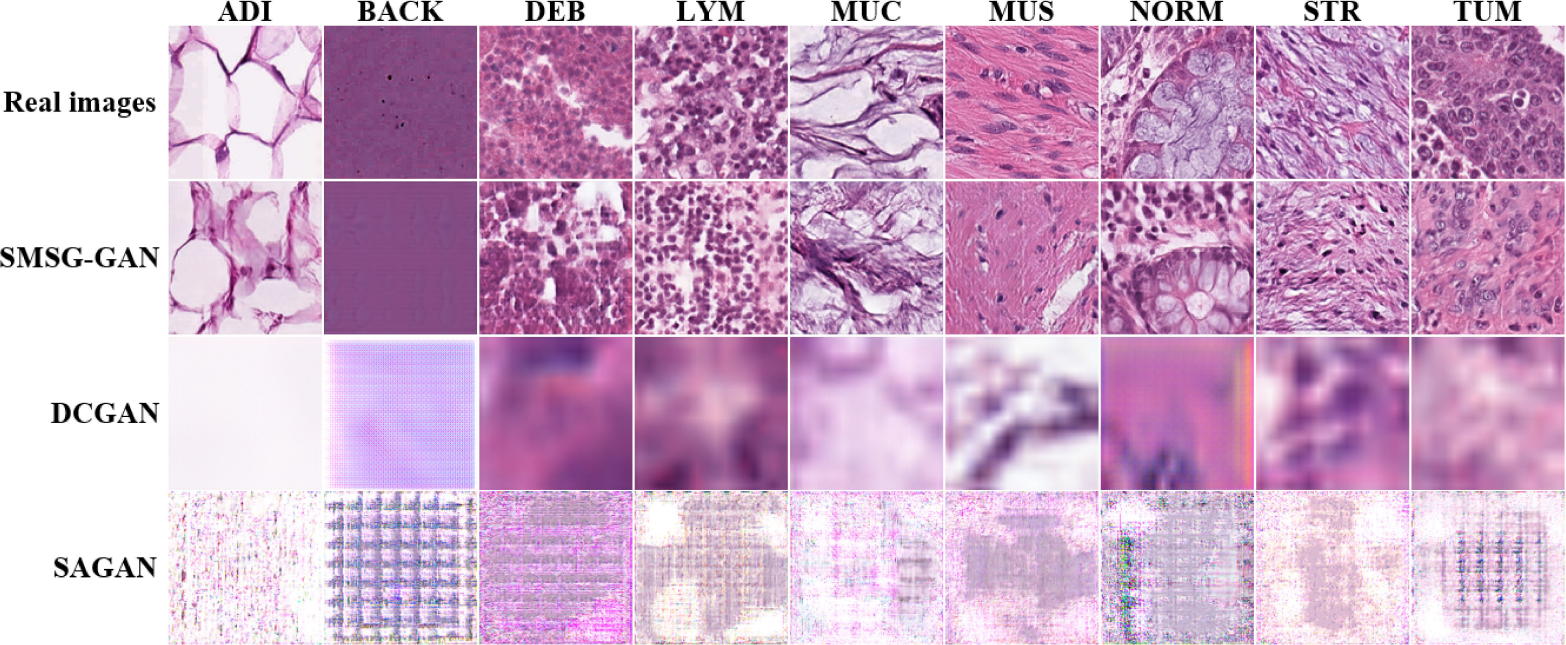
Results of synthetic colorectal tissue images for each class with different GANs.

**Figure 8** shows some of the samples for each class of synthesized colorectal tissue patches with different GANs. The first column represents the real image from the training data, and the remaining columns represent synthesized images by SMSG-GAN, DCGAN, and SAGAN, respectively. The images SMSG-GAN generated were enough to achieve a real effect compared with the other two GAN models. By contrast, the quality of the images DCGAN and SAGAN synthesized were both extremely unrealistic. The reason might be that their simple network architecture could not learn the features of large-scale images completely.

### 3.3 Ablation study of selective mechanism

To further analyze our proposed SMSG-GAN, we performed an ablation study of the selection mechanism and compared different thresholds used in the selection mechanism. We used the same backbone InceptionV3 classifier with the same hyperparameters setting in all experiments to make sure differences only come from the selection mechanism. In **Table 3**, *α* = 0 denoted that we utilized the synthetic images directly without selection for downstream classification experiments. *α* = 0.7 indicated that the generated images with a class probability higher than 0.7 were added to the original data for further classification. The overall accuracy of classification is illustrated in **Table 3**. One can see that either too small or too large a value of *α* compromises the advantage of selection. InceptionV3-SMSG-GAN with threshold *α* = 0.85 achieved the best performance. Data augmentation can improve classification performance, with better enhancement achieved when using the selection mechanism. However, the generated images have different levels of quality. The diversity decreases if a higher threshold is chosen, which results in no significant performance improvement.

**Table 3:** Classification results of different thresholds in selection mechanism.

α	0	0.7	0.85	0.95
**Overall Accuracy**	87.72%	87.24%	**89.54**%	86.87%

The bold values indicate the best performance.

### 3.4 Quantitative evaluation of generated images

In order to compare the three GAN models more comprehensively, we used three evaluation metrics to measure the quality and diversity of the synthesized images. The details of the three metrics are as follows.

*•* Inception Score(IS). The metric is widely used in GANs, the higher IS is, it indicates that the generator can generate high-quality samples (35). However, IS also has serious limitations. It is primarily to ensure that the samples generated by the model can be confidently identified as belonging to a specific class and that the model generates samples from multiple classes, not necessarily to assess the authenticity of the details or the diversity within the class. In other words, IS does not penalize a lack of intra-class diversity, Specifically, if mode collapse occurs in the generator, the value of the IS might be pretty, but the real situation is very bad. Thus, it is not sufficient to only use IS to evaluate the quality of the generated images.
*•* Frechet inception distance(FID). The FID is used to detect the intra-class mode dropping. It has been shown to be better aligned with expert evaluation in assessing the realism and diversity of the synthesized images (32). The metric captures the similarity of generated images to real ones, computing the distance between the feature vector of generated and real images. Usually, a synthesized image with good quality should result in a lower FID score.
*•* Kernel Inception Distance(KID). However, the IS and FID cannot well process the overfitting problem (36). To address this problem, the KID was proposed. The KID can capture higher order statistics and has an unbiased estimator but has been empirically found to suffer from high variance, which is a metric similar to the FID (36). Therefore, we also used KID to evaluate the quality of the synthetic images. The same as the FID, a lower value means better quality of synthesized images.

The three metrics mentioned above take a list of feature vectors extracted from images to compute the distance between distributions. The image feature vectors are extracted by the pre-trained InceptionV3 model trained on ImageNet (37). ImageNet is a large-scale database that is much larger in size and diversity and much more accurate, containing 12 subtrees with 5247 sets and 3.2 million natural images in total. Although the distribution of natural images and that of histopathology images are considerably different, the evaluation metrics may be more accurate when the pre-trained model used in the metrics train in a tremendous amount of data set and the data set used in our experiment is relatively tiny compared to ImageNet. Hence we eventually did not replace the InceptionV3 model pre-trained on ImageNet with the same model pre-trained on the NCT-CRC-HE-100K data set.

**Figure 9:**
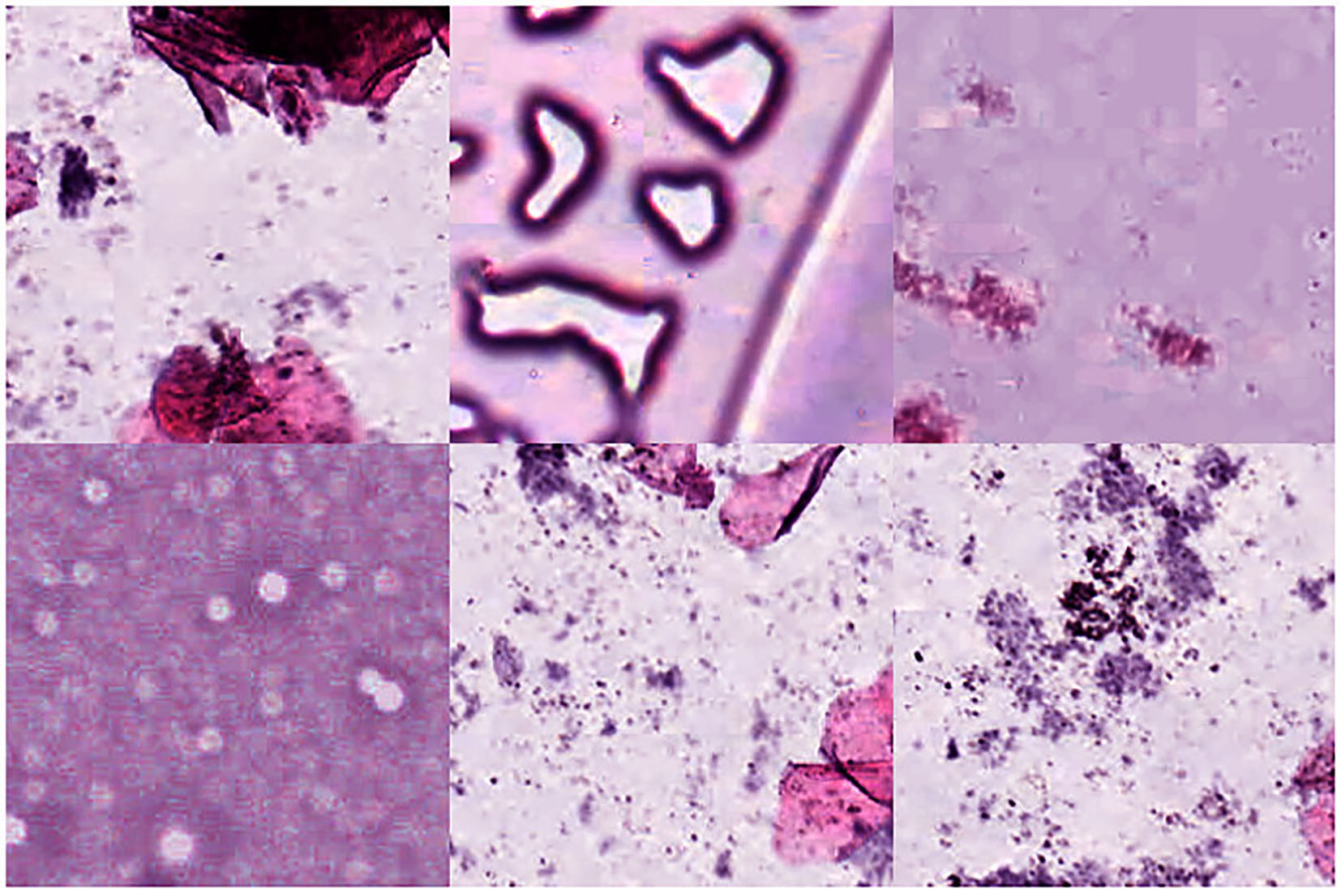
Samples from the BACK class in the NCT-CRC-HE-100K data set.

The results of the reference-based metrics applied to the evaluation of the synthesized images are listed in **Table 4**. We additionally utilized the additional two GAN models training on the same data set to generate tissue images for comparison. As shown in **Table 4**, In terms of IS, the values in SAGAN were the lowest. However, the values of IS in these three GAN models were highly close, which indicated that it was not accurate to evaluate image quality only with IS to some extent. In terms of the metric of FID and KID, SMSG-GAN yielded lower values except for the BACK class compared with DCGAN. Besides, the FID and KID values of the BACK class were much larger than those of the other classes in SMSG-GAN. We assumed that the BACK class was so complicated and didn’t follow any specific mode. Therefore, SMSG-GAN cannot fully learn its distribution. Some randomly selected images of the BACK class in original training data are displayed in **Figure 9**. SAGAN performed worst in these three metrics with the lowest IS, the highest FID and KID. Taken together, SMSG-GAN clearly outperformed the other two GAN models.

**Table 4:** The comparison of quantitative metrics of the proposed model with DCGAN and SAGAN.

	SMSG-GAN				DCGAN			SAGAN	
	IS *↑*	FID *↓*	KID *↓*	IS *↑*	FID *↓*	KID *↓*	IS *↑*	FID *↓*	KID *↓*
ADI	1.06	74.09	0.07	1.06	371.42	0.43	1.02	384.72	0.44
BACK	1.06	316.14	0.3	1.07	256.72	0.24	1.1	409.42	0.53
DEB	1.07	56.05	0.04	1.09	451.3	0.61	1	315.85	0.39
LYM	1.03	76.97	0.09	1.08	469.42	0.68	1.01	367.01	0.44
MUC	1.06	88.09	0.09	1.06	360.3	0.4	1.03	350.11	0.37
MUS	1.06	57.13	0.04	1.08	403.53	0.48	1	389.03	0.48
NORM	1.07	72.56	0.08	1.06	455.62	0.59	1.02	442.18	0.54
STR	1.05	66.35	0.06	1.04	396.68	0.53	1	311.55	0.39
TUM	1.07	32.14	0.03	1.04	428.99	0.59	1.02	359.47	0.39

## 4 Discussion

In this study, we proposed a novel method to improve classification for colorectal tissue images. A simple CNN classifier trained on the NCT-CRC-HE-100K data set without any synthesized images reached only 86.87% overall accuracy. when the images generated by SMSG-GAN were included in the original training data, overall accuracy was significantly improved to 89.54%. We used SMSG-GAN to generate colorectal tissue images with higher fidelity and fewer artifacts compared to other GANs. As an additional image quality verification, we tested synthetic images using three metrics. Lower values in FID and KID metrics SMSG-GAN got, compared with DCGAN and SAGAN, which demonstrated that our approach was able to capture real image features and more similar to the real data. In particular, we synthesized the nine classes of tissue patches separately. For further improvement, we applied a selection mechanism to filter the generated images that have ambiguous class labels, ensuring the selected images can conduce to the classification performance. In general, SMSG-GAN can generate more realistic images, and the generated images can be used as a training set for classification tasks, further improving the classification performance of colorectal cancer tissue images.

A data augmentation strategy was proposed to enhance the performance of tissue classification, thereby assisting pathologists in accurately diagnosing colorectal cancer cases. In practice, the training of CNNs requires an abundant supply of data. However, factors such as privacy concerns and the high cost of labeling result in limited annotated data. The proposed data augmentation strategy addresses this issue by providing training samples that closely resemble real data.

Some limitations that need to be addressed in further research are as follows. First, we did not generalize our method to other datasets. Second, the SMSG-GAN model in our method was complex, it was quite a time-consuming process. Hence, a possible direction for future work is to find an efficient way to lighten the model. Besides, we will do more research about training parameters to further enhance model performance. Also, we will explore other variants of GAN models as a data augmentation approach to improving the overall performance of deep networks and applying it to other biomedical datasets.

## Data availability statement

Publicly available datasets were analyzed in this study. This data can be found here: https://www.zenodo.org/record/1214456#.YsZJoGBBxPY.

## Author contributions

Conceptualization, ZL, JZ, WC, LJ, and SH; Formal analysis, ZL and SH; Methodology, LJ and SH; Data curation, WC, LJ, and CL; Investigation, LJ and SH; Resources, SH; Writing—original draft preparation, SH; Writing—review and editing, ZL, JZ, LJ, and SH; Visualization, LJ and SH; Supervision, ZL and JZ; Project administration, LJ and SH. All authors contributed to the article and approved the submitted version.
